# Classification of videolaryngoscopy: a systematic review

**DOI:** 10.1111/anae.70358

**Published:** 2026-08-26

**Authors:** Catharina Grün, André Dankert, Viktor A. Wünsch, Yasmin Engels, Aljoscha Ewert, Hans‐Heinrich Sieg, Lili Plümer, Christian Zöllner, Martin Petzoldt

**Affiliations:** ^1^ Department of Anaesthesiology, Centre for Anaesthesiology and Intensive Care Medicine University Medical Centre Hamburg‐Eppendorf Hamburg Germany

**Keywords:** airway management, classification, documentation, intubation, intratracheal, laryngoscopy

## Abstract

**Introduction:**

Structured classification and consistent documentation of videolaryngoscopy findings are essential for future airway planning. Although numerous classification tools for communication have been proposed, their accuracy and effectiveness remain uncertain. We aimed to synthesise the evidence on the accuracy, performance and inter‐rater reliability of existing tools used to classify videolaryngoscopic tracheal intubation.

**Methods:**

Databases were searched for original articles framed by the Participants – Index test – Target conditions criteria. We extracted accuracy, performance, concordance and reliability to evaluate the generalisability and clinical utility of each classification. We applied the QUADAS‐2 tool for quality assessment.

**Results:**

We identified 13 eligible studies evaluating characteristics of seven distinct classification tools in adults and children, including the Cormack–Lehane classification; percentage of glottic opening score; intubation difficulty scale; Fremantle score; video classification of intubation score; videolaryngoscopic intubation and difficult airway classification (VIDIAC) score; and paediatric difficult airway classification (PeDiAC) score. Eight publications reported clinical studies in patients. Since many studies reported only inter‐rater reliability or concordance, the accuracy, performance, generalisability and clinical utility of the respective classification tools remained uncertain. Accuracy or performance metrics were reported for three classification tools – the Cormack–Lehane classification, VIDIAC and PeDiAC scores – with the first showing only limited performance. Empirically‐derived thresholds were not reported for most classification tools. Overall risk of bias was high among the reviewed studies, particularly regarding the reference standard, flow and timing, accompanied by substantial applicability concerns.

**Discussion:**

Despite widespread dissemination of various proposed classification tools, current evidence remains limited. Accuracy, performance and thresholds have been evaluated for only three classification tools, while remaining largely unknown for the other four tools. Further high‐quality research is needed.

## Plain Language Summary

**What we did:**

Researchers looked at studies that tested different ways of describing how easy or hard it was for doctors to place a breathing tube into the patients' windpipe using a videolaryngoscope, a special tool with a camera at the tip. They reviewed 13 studies that examined seven different scoring systems and compared how accurate, reliable and useful they were for adults and children.

**Why did we do it:**

Doctors need a clear and reliable way to record how difficult videolaryngoscopic intubation was so that future airway care can be planned safely. Although several scoring systems have been created, it was not clear which ones work best or whether different doctors would give the same score in the same situation.

**What we found:**

The researchers found that there is only limited evidence showing how well these scoring systems work. Most studies looked only at whether different doctors agreed on the scores, rather than whether the scores themselves accurately predicted difficulty or helped improve communication and patient care. Only three of the seven scoring systems had been tested properly; two among them performed well and one only modestly. Overall, the quality of the available research was low, so more high‐quality studies are needed to identify the best way to describe and record videolaryngoscopy findings.

## Introduction

Videolaryngoscopy is used increasingly and, in general, has replaced direct laryngoscopy across several clinical applications and settings [1, 2, 3, 4, 5, 6]. Difficult videolaryngoscopic tracheal intubation is a major contributor to anaesthesia‐related adverse events [7, 8, 9, 10, 11, 12].

A history of difficult airway management is among the strongest predictors of future airway difficulties [13, 14, 15]. Medical documentation denotes the structured recording of clinical information in health records to ensure continuity of care among healthcare professionals across multiple encounters [16, 17, 18]. An increasing number of tools for classifying videolaryngoscopic tracheal intubation have been proposed to standardise medical documentation and to facilitate communication among airway operators through structured codes, scales, grades or scores [19, 20, 21, 22, 23, 24, 25]. The way these tools are used directly influences medical care and patient outcomes [18, 21, 22, 26] by supporting clinicians to anticipate risks, improve situational awareness and prevent airway‐related complications during future airway management [15]. As digitalisation advances, documented airway reports become more widely available [15, 16, 17].

Descriptive classification tools provide non‐interpretive documentation only and are not intended for clinical communication, decision‐making or care planning in a clinical context (e.g. forensic recordings, quality assessment, education or research). Any tool that records a characteristic, observation, condition or score of an individual to infer something about the health state at that timepoint is a medical test that necessitates evaluation of its accuracy before being considered clinically applicable [19, 20, 21, 22, 23, 27]. Beyond adequate accuracy, tools to classify videolaryngoscopic tracheal intubation must also show clinical utility and generalisability to be effective [19, 20, 21, 22, 25, 28] (Table 1). Clinical utility requires deriving rational thresholds to balance sensitivity and specificity and support meaningful severity grading (empirical thresholds) [21, 29]. Using classification tools with unknown or poor accuracy can lead to over‐ or undertreatment, while high accuracy can improve patient outcomes by enabling more effective interventions [15, 21, 22, 26, 27, 29].

**Table 1:** Terminology and definitions used in the review to assess classification tools.

	Definition
Classification tool	Any medical test that uses a characteristic, observation, condition or score to infer something about the health state at that timepoint; provides categories or numerical scales; and is intended to be used for clinical documentation and communication.
Accuracy	Ability to detect the condition that the tool is intended to assess, i.e. to discriminate between individuals with and those without that condition using sensitivity and specificity. Assessment of accuracy requires a threshold and reference standard.
Performance	Ability to discriminate between individuals with and without a condition across all possible thresholds. Usually summarised by the area under the receiver operating characteristic curve.
Thresholds	Distinguish between normal and abnormal findings.
Calibration	Agreement between the observed and predicted outcome, illustrated frequently as calibration curve or belt.
Clinical utility	Measurable positive effect of the tool on the health state of patients. Clinical utility requires accuracy and thresholds to allow appropriate clinical interpretation and to support treatment decisions.
Generalisability/validation	Ability to provide accurate classifications in a new sample of patients. In development and validation studies this is usually assessed by internal validation (i.e. resampling or cross‐validation) or external validation (new sample).
Concordance	Concordance or agreement between raters refers to the degree to which different raters provide the same rating when using the same tool in the same patient. Often expressed as proportion of identical ratings across raters.
Reliability	Degree to which the results can be replicated under different conditions. Inter‐rater reliability refers to the agreement between two or more raters, intra‐rater reliability to the agreement between repeated observations of the same rater, in the same subject, using the same tool.

Various tools to classify videolaryngoscopic intubation exist, such as the Cormack–Lehane classification and its modifications [30, 31, 32]; percentage of glottic opening (POGO) score [33]; intubation difficulty scale [34]; Fremantle score [35]; video classification of intubation (VCI) score [36, 37]; videolaryngoscopic intubation and difficult airway classification (VIDIAC) score [11, 38]; and paediatric difficult airway classification (PeDiAC) score [39]. However, test characteristics, such as accuracy, performance, generalisability and inter‐rater reliability of each remain uncertain.

This systematic review aimed to synthesise the current scientific literature on the accuracy, performance and inter‐rater reliability of existing tools to classify videolaryngoscopic tracheal intubation across diverse medical contexts.

## Methods

This systematic review of the literature was conducted in accordance with PRISMA [40], PRISMA of diagnostic test accuracy studies [41, 42] and STARD [19, 20] statements. We used R‐AMSTAR criteria for internal audit of the review [43]. We aimed to identify eligible studies and provide a qualitative and quantitative synthesis of extracted data, as well as a structured quality assessment of the existing scientific literature. To avoid redundancy, we searched PROSPERO and the Cochrane Library for related systematic reviews before we registered the systematic review protocol with PROSPERO [44].

Participants – Index test – Target conditions (PIT) criteria were applied for framing the review question (online Supporting Information Table S1) [21, 42]. Prospective or retrospective original research studies involving adult or paediatric patients that evaluated accuracy, performance, concordance or inter‐rater reliability of classification tools were included. Any clinical setting was eligible, including operating theatre; ICU; emergency department; paediatrics; or training environments. Studies that employed existing classification tools solely as baseline characteristics or outcome measures, without evaluating their test characteristics, were not included. Studies conducted on manikins or cadavers were also not included. Post‐hoc analyses of image or video material were included if the source was originally human‐derived, even if the study lacked a cohort design, clinical context or direct patient involvement. Correspondence or brief scientific reports were deemed eligible if relevant original research data were presented and methods were reported clearly. Only studies published in the English language were considered.

We specified that the index test could be any tool used to classify any representation of difficult videolaryngoscopic tracheal intubation, such as models; scores; grades; classes; scales; or a single predictor variable assessed during or directly after airway management. In contrast, prognostic tools (assessed during pre‐operative evaluation) that estimate the probability that an event will occur in the future (e.g. to facilitate pre‐operative risk prediction) were not within the scope of this review [21].

We systematically searched MEDLINE, CINAHL and the Cochrane Central Register databases for potentially eligible studies up to 12 December 2025 (online Supporting Information Table S2) [44]. Additional sources such as journal websites, systematic reviews, guidelines or narrative review articles, as well as the reference lists from the identified original articles, were further checked for eligible articles. Identified studies were uploaded into EndNote 21 (Clarivate Analytics, London, UK) to facilitate a structured review and deduplication. Title, abstract and full text screening, as well as data extraction, were performed by two independent authors, with adjudication by a third if required.

Relevant findings from included studies were summarised using a narrative, qualitative synthesis. Pooling of data (sensitivity and specificity) for the purpose of a structured quantitative data synthesis and meta‐analysis was planned for any classification evaluated in at least three eligible studies that reported accuracy [19, 20, 41, 42], depending on the degree of heterogeneity (design, exposure, population, outcome definition or methodology) across the identified studies [45]. We planned to use random‐effects models for quantitative data synthesis to compensate for unexplained heterogeneity. A narrative synthesis of relevant test characteristics of the evaluated classifications was planned if a meta‐analysis was not feasible, in accordance with the PRISMA [40, 41, 42] and STARD [19, 20] recommendations.

We extracted relevant test characteristics such as accuracy, performance, concordance and inter‐rater reliability from all identified studies across different tools, settings and populations (Table 2 and online Supporting Information Table S3).

Accuracy, expressed by sensitivity and specificity [21, 22], was the primary outcome of interest in this systematic review. Before determining whether a tool is generalisable and effective in improving patient outcomes, it is necessary to determine whether it can be used to accurately classify the target condition [22]. We further extracted performance metrics of the tools, given by the area under the receiver operating characteristic curve (AUROC) with 95%CI [21, 29, 46, 47, 48] as well as threshold values, which are relevant for clinical utility by distinguishing normal from abnormal findings [21, 47]. We considered AUROC values of 0.51–0.69 to indicate poor; 0.70–0.79 to indicate fair; and ≥ 0.80 to indicate good performance [49].

The calibration of a classification tool refers to the agreement between the predicted and observed outcome [48, 50]. Calibration plots are used to assess calibration. We considered the calibration to be good if the calibration plot showed strong agreement between the predicted and observed outcomes across the full range of probabilities without relevant over‐ or underestimation [29, 47, 50].

**Table 2:** Studies evaluating characteristics of classification tools for videolaryngoscopy.

	Studies*	Accuracy	Performance	Calibration	Empirical thresholds	Inter‐rater reliability
Cormack–Lehane classification; adults	2 clinical 4 post‐hoc	–	Fair AUROC 0.75 [11]	–	–	Inconclusive [51, 52, 53, 54, 55] Cohen's κ or Fleiss' κ 0.21–0.77; concordance 57–75%
Cormack–Lehane classification; children	1 clinical 1 post‐hoc	–	Poor AUROC 0.68 [5]	–	–	Moderate Fleiss' κ 0.55 [5]; concordance 100% in neonates [56]
POGO score	2 post‐hoc	–	–	–	–	Moderate Cronbach's α 0.61; concordance 76% [55]; intraclass correlation coefficient 0.72; concordance 19% [51]
Intubation difficulty scale	1 clinical	–	–	–	–	–
Fremantle score	1 post‐hoc	–	–	–	–	Good Fleiss' κ 0.62; concordance 74% [55]
VCI	2 clinical	–	–	–	–	Good Concordance 97% [36] and 89% [37]; Krippendorff's α 0.86 [37]
VIDIAC score; adults	2 clinical	Sensitivity 0.74; specificity 0.95 [11]; and sensitivity 0.82; specificity 0.68 [38] (2 pts.^#^)	Good AUROC 0.92 [11] and 0.93 [38]	Good [11, 38]	Yes [11]	Good Fleiss' κ 0.72–1.0 [11]
VIDIAC score; children	1 clinical	Sensitivity 0.68; specificity 0.96 [39] (0 pts.^#^)	Good AUROC 0.80 [39]	Good [39]	Yes [39]	–
PeDiAC score	1 clinical	Sensitivity 0.91; specificity 0.93 [39] (2 pts.^#^)	Good AUROC 0.97 [39]	Good [39]	Yes [39]	–

POGO, percentage of glottic opening; VCI, video classification of intubation; IDS, intubation difficulty scale; VIDIAC, videolaryngoscopic intubation and difficult airway classification; PeDiAC, paediatric difficult airway classification; AUROC; area under the receiver operating characteristic curve; *clinical studies in patients or post‐hoc analysis of image or video material; ^#^respective threshold value.

To approximate generalisability [19, 20, 21, 25], which refers to the ability to provide accurate predictions in a new sample of patients [25], we recorded the number and types of studies for each classification tool investigated. For multivariable models, we report the level of validation, which refers to performance at multiple stages, at model development (internal validation using resampling methods) and in new data sets (external validation) to assess generalisability across clusters and settings [29, 47, 48, 57].

Reliability refers to the agreement between two or more observations of the same entity by different (inter‐rater) or the same (intra‐rater) raters using the same classification tool [24, 26, 58, 59]. While assessment of inter‐rater reliability is a relevant methodological step when examining whether a given tool can be applied consistently across raters, it does not directly indicate whether the tool is effective or accurately classifies the condition in question [19, 22, 24, 51, 58]. We expressed the concordance between raters as the proportion of ratings where one rater agrees with the rating of another rater or group of raters (%) [26, 58, 59, 60]. Reliability between different raters was calculated as weighted Cohen's κ, Fleiss' κ or Krippendorff's α for categorical variables and intraclass correlation coefficient for continuous variables. Cronbach's α measures internal consistency, indicating how closely related a set of items is. In this review, κ values < 0.20 were considered to indicate poor; 0.21–0.4 fair; 0.41–0.60 moderate; and > 0.61 good agreement among raters [60, 61]. Krippendorff's α values < 0.67 were considered to indicate poor; 0.67–0.79 moderate; and ≥ 0.80 good agreement among raters [62]. Intraclass correlation coefficient values < 0.5 were considered to indicate poor; 0.5–0.75 moderate; and 0.75–0.9 good reliability [63]. A Cronbach's α < 0.6 was considered to indicate poor; 0.6–0.8 moderate; and > 0.8 good internal consistency [64, 65].

We used the quality assessment of diagnostic accuracy studies – version 2 (QUADAS‐2) tool for quality assessment [21, 28, 66]. This evaluates the risk of bias and applicability concerns for each classification tool in the included studies using signalling questions across four domains (patient selection, index test, reference standard and flow and timing) and ranks each domain as either low, high or unclear risk of bias and applicability concerns [28]. As many studies investigated more than one classification, and some adopted differing methodologies for different classifications, we present the overall risk of bias and applicability concerns for each investigated classification in each clinical study separately. For each included study, three reviewers independently assessed the risk of bias and applicability concerns using a structured checklist and template. Quality assessment was further supplemented by the quality appraisal tool for studies of diagnostic reliability (QAREL) [24] for studies investigating the inter‐rater reliability and prediction study risk of bias assessment tool (PROBAST) [67, 68] for model development and validation studies.

## Results

We identified 1858 publications through our search strategy and four additional ones by hand searching; 13 were included in the analysis [5, 11, 36, 37, 38, 39, 51, 52, 53, 54, 55, 56, 69] (Fig. 1). Ten predominantly included adults [11, 36, 37, 38, 51, 52, 53, 54, 55, 69] and three solely children [5, 39, 56] (Table 2 and online Supporting Information Table S3). Four studies examined test characteristics of more than one classification [11, 39, 51, 55]. Two reported from the same prospective cohort [5, 39] and two others appeared to overlap in the studied populations [53, 54].

**Figure 1:**
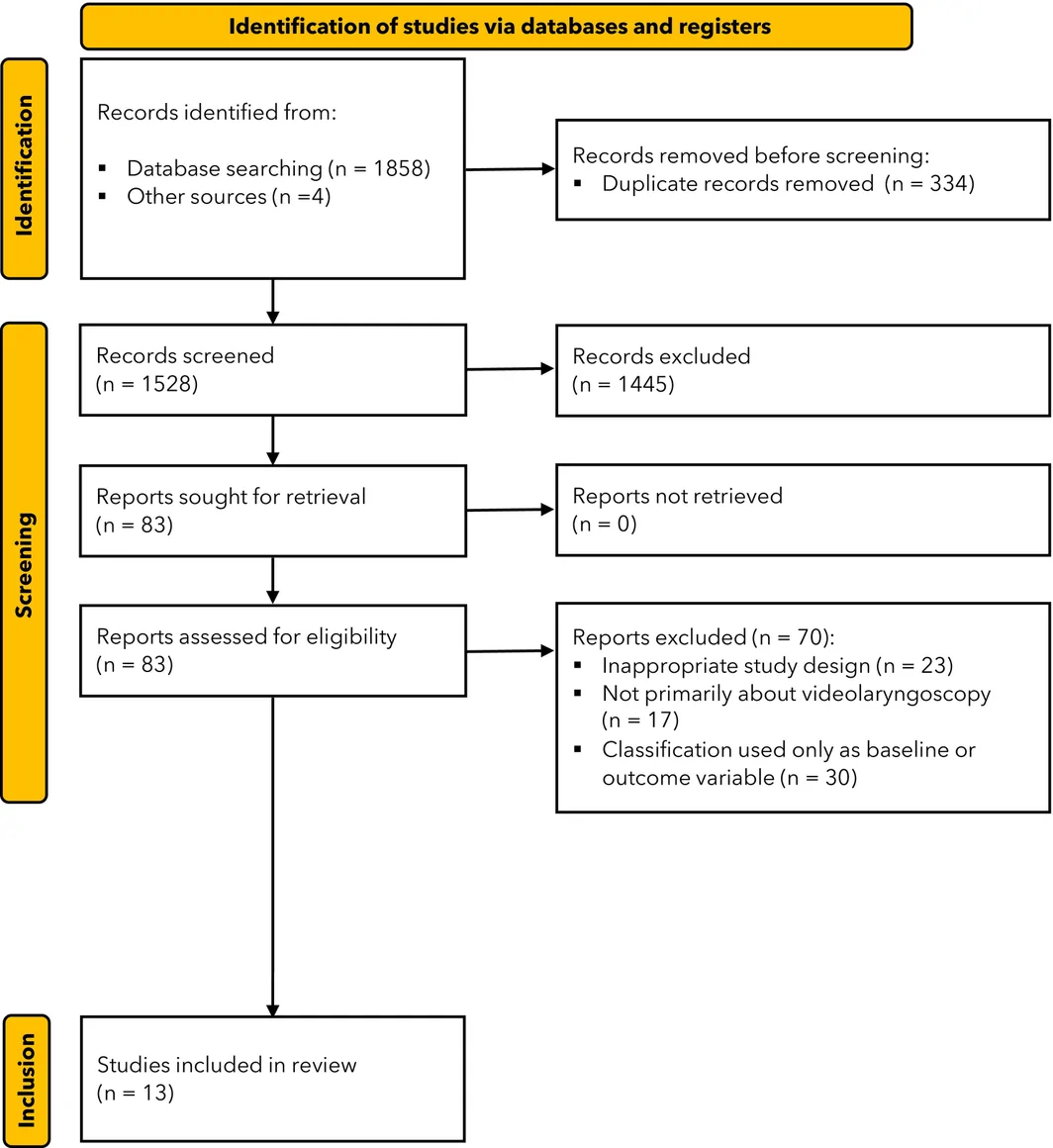
Study flow diagram.

Eight articles report clinical studies in patients [5, 11, 36, 37, 38, 39, 54, 69] (Table 2), including six prospective cohort studies [5, 11, 36, 37, 38, 39], one retrospective cohort study [54] and one post‐hoc analysis of pooled data [69]. Among these, four provided accuracy or performance metrics [21] of three distinct classification tools [5, 11, 38, 39]. Another five report post‐hoc analyses of human‐derived images or videos [51, 52, 53, 55, 56] (online Supporting Information Table S3). Most studies did not define thresholds [5, 36, 37, 51, 53, 55, 69]; three used a pre‐specified [52, 54, 56]; and two determined empirical thresholds based on sensitivity, specificity, Youden index or decision curve analysis [11, 39]. Seven studies [36, 37, 51, 52, 54, 55, 56] assessed reliability and/or concordance between raters or groups of raters without assessing accuracy or performance [19, 21, 22, 25].

Two studies investigating the Cormack–Lehane classification showed fair performance in adults (AUROC 0.75, 95%CI 0.70–0.80) [11] and poor performance in children (AUROC 0.68, 95%CI 0.59–0.78) [5]. A retrospective cohort study found fair inter‐rater reliability between paramedics/nurses and post‐hoc video‐based physician ratings [54]. Numerous post‐hoc video/image analyses [51, 52, 53, 55, 56] reported partly inconclusive findings regarding the inter‐rater reliability, ranging from fair to good across different populations (e.g. adults, neonates and emergencies) and rater groups (anaesthetists, critical care physicians and flight crew members).

The intubation difficulty scale was evaluated for videolaryngoscopy in a post‐hoc analysis of pooled data [69], showing an association with tracheal intubation difficulty (rated on visual analogue scales, correlation coefficients 0.70) and tracheal intubation time (correlation coefficients 0.54). Thresholds were not defined, and diagnostic accuracy and performance not assessed.

We did not find studies reporting accuracy metrics such as sensitivity, specificity or performance of the POGO score, Fremantle score or VCI [35, 36, 37, 51, 55]. The POGO score was assessed in two post‐hoc video/image analyses that found moderate inter‐observer reliability among anaesthetists and critical care physicians [51, 55]. One study compared images from Macintosh with hyperangulated videolaryngoscopy in identical subjects to evaluate POGO scores, referred to as “*correct POGO*” by the authors. The mean bias (standard deviation) between individual POGO ratings and this “*correct POGO*” was 13% (21%) in the Bland–Altman plot [51]. A large multicentre feasibility study assessed five pre‐specified POGO score categories (0%; 25%; 50%; 75%; 100%) but did not report sensitivity, specificity or performance [37]. The Fremantle score showed good inter‐rater reliability between 74 critical care physicians and four anaesthetists in a post‐hoc video analysis, but has not yet been evaluated in patients [55]. For the VCI score, a prospective cohort study and a multicentre feasibility study found good inter‐rater reliability between two staff members [36, 37]. Empirical thresholds have not been evaluated for the VCI, Fremantle score or POGO score [35, 36, 37, 51, 55].

The PeDiAC score originates from a prospective cohort study indicating good accuracy, performance (AUROC 0.97, 95%CI 0.96–0.99) and calibration [39]. It has been validated internally [39] but inter‐rater reliability has not been evaluated. Thresholds were derived empirically; ≥ 2 points (highest Youden index) define difficult videolaryngoscopic tracheal intubation in children.

The VIDIAC score originates from a prospective cohort study indicating good accuracy, performance (AUROC 0.92, 95%CI 0.89–0.95) and calibration [11]. Each of the six score parameters achieved good inter‐rater reliability. Thresholds were derived empirically by decision curve analysis and were defined as follows: ≤ 0 points, easy; 1 point, moderate; 2 points, hard; and ≥ 3 points, severe videolaryngoscopic tracheal intubation. Findings were confirmed across diverse populations: internal validation [11]; external validation with different blades (AUROC 0.93, 95%CI (0.88–0.97)) [38]; and in children (AUROC 0.80, 95%CI (0.73–0.87)) [39].

Quality assessment using the QUADAS‐2 tool revealed a high risk of bias among the included studies, particularly regarding the reference standard (17/18, 94%) and flow and timing (10/18, 56%), accompanied by substantial applicability concerns (Fig. 2 and online Supporting Information Figure S1). Post‐hoc video analyses carry a potential risk of non‐consecutive inclusion [51, 52, 53, 55, 56]. Rating videos under experimental conditions is artificial and likely produces overoptimistic results, as contextual factors, cognitive load, human factors and limited rating time cannot be considered. Additional quality assessments for studies evaluating inter‐rater reliability and for model development and validation studies are reported in online Supporting Information Tables S4 and S5.

**Figure 2:**
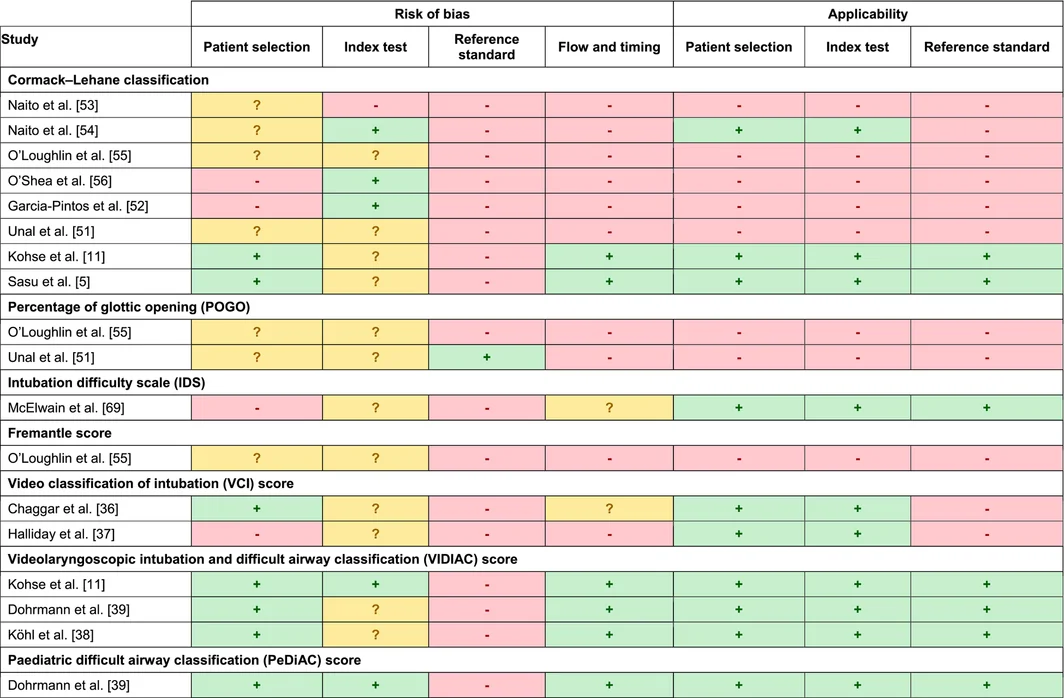
Quality assessment using the QUADAS‐2 tool showing the rater judgement about the risk of bias and applicability concerns for each classification in each included study. Green boxes, low; red boxes, high; amber boxes, unclear risk of bias or applicability concerns.

## Discussion

Although various classification tools for videolaryngoscopy have been proposed, our systematic review shows that current evidence remains limited. We identified 13 studies that investigated seven distinct tools to classify videolaryngoscopic tracheal intubation. The overall risk of bias across the studies was high. For many classification tools, accuracy, performance, thresholds and generalisability have not been investigated.

Classification tools use codes, scales, grades or scores to infer tracheal intubation conditions with videolaryngoscopes and require evaluation of accuracy before they can be used reliably for documentation, communication and exchange of findings among healthcare professionals [19, 20, 21, 22, 23, 27, 42]. High accuracy of a tool can potentially improve patient outcomes by enabling more rational decision‐making and effective interventions [15, 21, 22, 26, 27, 29]. The clinical utility of such tools relies on clinically meaningful thresholds to distinguish normal from abnormal findings and form the basis for severity grading [21, 29]. However, for many tools, empirical thresholds have not been determined [5, 36, 37, 51, 53, 55, 69].

The accuracy or performance has been reported for three classification tools: the Cormack–Lehane classification; VIDIAC score; and PeDiAC score. The Cormack–Lehane classification showed fair performance in adults and poor performance in children [5, 11]. The PeDiAC score performed well in children [39] and the VIDIAC score performed well in adults [11, 38] and children [39]. The generalisability in new populations was evaluated for the Cormack–Lehane classification and VIDIAC score [25]. For the POGO score, intubation difficulty scale, Fremantle score and VCI, accuracy metrics, such as sensitivity, specificity or performance have not been investigated [36, 37, 51, 55, 69], leaving their clinical utility and generalisability unknown [20, 21, 22, 25, 41, 42].

Many studies that evaluated the Cormack–Lehane classification, POGO score, Fremantle score or VCI solely assessed reliability and/or concordance between individual raters and either a second rater or a panel of raters [36, 37, 51, 52, 53, 54, 55, 56]. Assessing inter‐rater reliability is a relevant methodological step when examining whether a given tool can be applied reliably across raters [16, 19, 21, 51, 58], as it reflects consistency between judgements from different observers. However, it does not indicate whether the classification itself is accurate or clinically useful [19, 21, 22, 25]. Reliability is clinically meaningful if assessed in conjunction with accuracy [24, 51, 58]. Reported inter‐rater reliability for the Cormack–Lehane score was partly inconclusive [51, 52, 53, 54, 55, 56]. Two clinical studies assessed inter‐rater reliability of the VCI between a staff member performing the tracheal intubation and another one observing the tracheal intubation done by the first staff member [36, 37]. Extrapolating this to clinical practice, where the classification is intended to support longitudinal communication across healthcare professionals for future airway planning, appears questionable [21].

Our systematic review of the literature has some limitations. We identified substantial heterogeneity among the reviewed studies regarding design; population; index tests; and reference standards. The large number of proposed classification tools, small number of studies per tool and heterogeneous study methodologies precluded qualitative synthesis. To handle heterogeneity among study designs, we applied the STARD and PRISMA‐DTA recommendations and the QUADAS‐2 tool, which provide overarching guidance for all types of tools and designs. In addition, we used the QAREL tool for studies evaluating inter‐rater reliability and the PROBAST tool for model development and validation studies. Many studies included had methodological flaws and a high risk of bias, particularly with respect to patient selection, as they relied on post‐hoc video or image reviews, or used non‐consecutive inclusion, potentially introducing selection and spectrum bias. Further, definitions of the target condition varied broadly across the reviewed studies.

In conclusion, despite widespread dissemination of various proposed tools to classify videolaryngoscopic tracheal intubation, current evidence remains limited. We identified 13 eligible studies reporting seven videolaryngoscopy classifications in adults and children. Many studies showed distinct methodological flaws, high risk of bias and substantial applicability concerns. Accuracy, performance and thresholds have been evaluated for three classification tools – the Cormack–Lehane classification, VIDIAC and PeDiAC scores – while remaining largely unknown for the other four tools. Further high‐quality research is needed.

## Supporting information


**Figure S1.** Quality assessment using the QUADAS‐2 tool.

**Table S1.** Participants–index test–target conditions criteria.
**Table S2.** Search strategies.
**Table S3.** Qualitative synthesis of studies.
**Table S4.** Quality appraisal using the QAREL checklist.
**Table S5.** Summary of risk of bias using the PROBAST instrument.
