# Supplementary material for: Classification of videolaryngoscopy: a systematic review
Source: Anaesthesia. 2026 Aug 26;81(12):1731–41. doi: 10.1111/anae.70358 (PMC13743675; doi:10.1111/anae.70358)
Supplement: Supplementary file 1 [file ANAE-81-1731-s001.pdf]

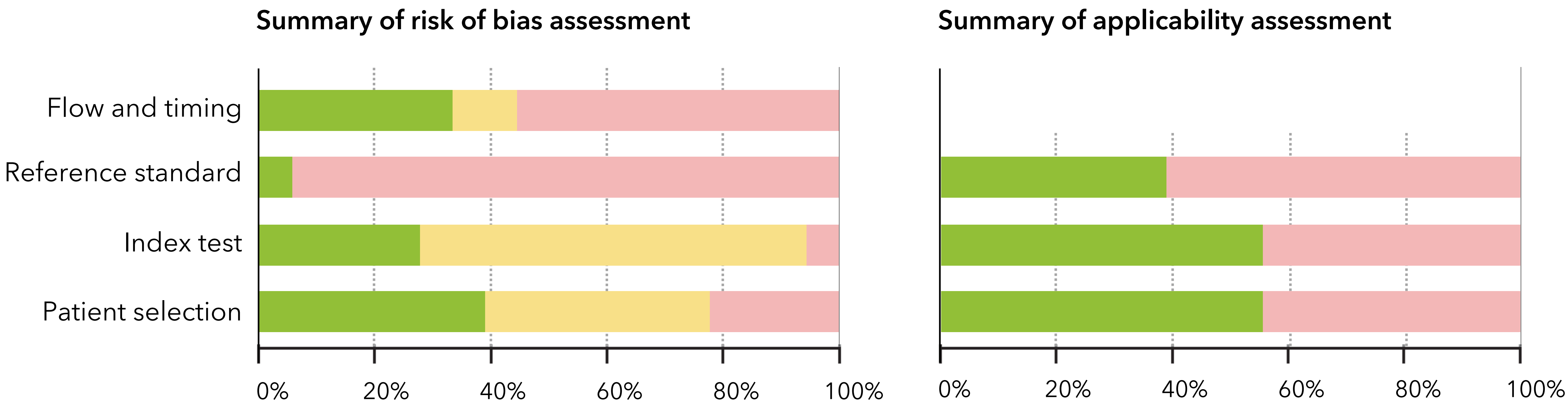

Figure S1 Quality assessment using the QUADAS-2 tool showing the summary of raters' judgements about the risk of bias and applicability concerns among all included studies (n=18).
Green bars "low"; red bars "high"; yellow bars "unclear" risk of bias (left) or applicability concerns (right), respectively.
