# Supplementary material for: Classification of videolaryngoscopy: a systematic review
Source: Anaesthesia. 2026 Aug 26;81(12):1731–41. doi: 10.1111/anae.70358 (PMC13743675; doi:10.1111/anae.70358)
Supplement: Supplementary file 2 [file ANAE-81-1731-s002.docx]

**Table S1** Participants – Index test – Target conditions (PIT) criteria for a priori framing of the review question.

| **Criterion** | |
| --- | --- |
| Participants | Individuals undergoing videolaryngoscopic tracheal intubation (children and adults without age restrictions) in various settings (e.g. operating theatre, ICU, ED, paediatrics or educational settings) |
| Index test | Models, scores, grades, classes, scales or single predictor variables used for classification during or directly after tracheal intubation |
| Target conditions | Various representations of difficult videolaryngoscopic intubation |

**Table S2** Final search terms for PubMed.

("videolaryngoscopy "[All Fields] OR "video laryngoscopy"[All Fields] OR "videolaryngoscope"[All Fields] OR "video laryngoscope"[All Fields] OR "videolaryngoscopic"[All Fields] OR "indirect laryngoscopy"[All Fields] OR “indirect laryngoscopes"[All Fields] OR “laryngoscopy video"[All Fields] OR “airway visualization"[All Fields] OR “laryngoscopists“[All Fields] OR “laryngoscopic exposure“[All Fields]) AND ("documentation"[All Fields] OR "prediction"[All Fields] OR "predict"[All Fields] OR "classification"[All Fields] OR "class"[All Fields] OR "grading"[All Fields] OR "grade"[All Fields] OR "scoring"[All Fields] OR "score"[All Fields] OR "scale"[All Fields] OR "model"[All Fields] OR "index"[All Fields] OR "laryngeal view"[All Fields] OR “laryngeal views“[All Fields] OR "larynx view"[All Fields] OR "glottic view"[All Fields] OR "glottis view"[All Fields] OR "glottic opening"[All Fields])

**Table S3** Qualitative synthesis of studies evaluating tools to classify videolaryngoscopic tracheal intubation grouped by the index test (classification).

| **Study** | | **Study design** | **Generalisability** | **Participants or data source** | **Index test (raters)** | **Target condition of the study** | **Outcome incidence n (%)** | **Inter-rater reliability, concordance between raters (%)** | **Thresholds** | **Performance (Perf.), Accuracy (Acc.), Calibration (Cal.)** |
| --- | --- | --- | --- | --- | --- | --- | --- | --- | --- | --- |
|  | **Cormack-Lehane classification (C/L)** | | | | | | | | | |
| Naito et al. 2016 [53] | | post hoc video analysis | - | 268 videos (304 attempts); air medical services; 15 videos for inter--rater reliability | post hoc C/L rated by one experienced physician | successful TI attempt (from videos); post hoc C/L rated by another physician | 244/304 (80%) | good-Cohen’s κ 0.77 | not defined | Perf.: not assessed│Acc.: not assessed│Cal.: not assessed│association: odds ratio 0.23 (0.12–0.45)^#^ |
| Naito et al. 2016 [54] | | retrospective, post hoc video analysis | - | 236 patients; air medical services | prehospital C/L rated by paramedics/ nurses (charts)^§^ | post hoc C/L rated by physicians | no outcome measure | fair-Cohen’s κ 0.21, concordance 57%; | set to: C/L 1-2 “easy”; 3-4 “difficult” | Perf.: not assessed│Acc.: not assessed│Cal.: not assessed |
| O'Loughlin et al. 2017 [55] | | post hoc video analysis | - | 25 videos, unknown setting | post hoc C/L rated by 74 critical care physicians | post hoc C/L rated by 4 senior anaesthetists* | no outcome measure | moderate-Fleiss κ 0.46, intra-rater 0.77, concordance 65% | not defined | Perf.: not assessed│Acc.: not assessed│Cal.: not assessed |
| O'Shea et al. 2018 [56] | | post hoc video analysis | - | 45 videos; neonates, unsuccessful TI, NICU | post hoc C/L rated by one physician | post hoc C/L rated by another physician | no outcome measure | concordance 100% | set to: C/L 1-2 vs. 3-4 | Perf.: not assessed│Acc.: not assessed│Cal.: not assessed |
| Garcia-Pintos et al. 2021 [52] | | post hoc video analysis | - | 10 videos; air med. services | post hoc C/L rated by 57 flight crew members | post hoc C/L rated by 3 physicians* | no outcome measure | moderate-Fleiss κ 0.44, concordance 72% | set to: C/L 1-2 vs. C/L 3-4 | Perf.: not assessed│Acc.: not assessed│Cal.: not assessed |
| Unal et al. 2021 [51] | | post hoc image analysis | - | 20 images, OR | post hoc C/L rated by 75 anaesthetists | post hoc C/L rated by unspecified experts* | no outcome measure | fair-weighted κ 0.33, intra-rater 0.74, concordance 75%; | not defined | Perf.: not assessed│Acc.: not assessed│Cal.: not assessed |
| Kohse et al. 2022 [11] | | prospective study | external validation | 320 adults (374 data sets), exp. difficult airways; OR | C/L rated by 81 anaesthetists +2 independent raters | difficult videolaryngo-scopic intubation | 183/374 (49%) | not provided | not defined | Perf.: fair-AUROC 0.75 (0.70–0.80)│Acc.: not assessed│Cal.: none |
| Sasu et al. 2024 [5] | | prospective study | external validation | 809 children (904 data sets); OR | C/L rated by anaesthetists^§^+1 independent rater | difficult videolaryngo-scopic intubation | 47/904 (5%) | moderate-Fleiss κ 0.55 | not defined | Perf.: poor-AUROC 0.68 (0.59–0.78)│Acc.: not assessed│Cal.: none |
|  | **Percentage of glottic opening (POGO)** | | | | | | | | | |
| O'Loughlin et al. 2017 [55] | | post hoc video analysis | - | 25 videos, unknown setting | post hoc POGO rated by 74 critical care physicians | post hoc POGO rated by 4 senior anaesthetists* | no outcome measure | moderate-Cronbach’s alpha 0.61, intra-rater 0.69, concordance 76% | not defined | Perf.: not assessed│Acc.: not assessed│Cal.: not assessed |
| Unal et al. 2021 [51] | | post hoc image analysis | - | 20 images, OR | post hoc POGO rated by 75 anaesthetists | “Correct POGO” assessed by HA-VL and MAC-VL images by expert panel | continuous (“correct POGO”) | moderate-ICC 0.72, intra-rater 0.78, concordance 19% | not defined | Perf.: not assessed│Acc.: not assessed│Cal.: not assessed│agreement: 13±21% between single raters and panel |
|  | **Intubation Difficulty Scale (IDS)** | | | | | | | | | |
| McElwain et al. 2011 [69] | | pooled data from prospective studies | - | 210 adults, pooled data from 6 studies, OR | IDS sampled in 6 studies^§^ | intubation difficulty on a VAS scale and intubation time (s) | continuous | not provided | not defined | Perf.: not assessed│Acc.: not assessed│Cal.: not assessed│correlation coefficients: intubation difficulty 0.70 and time 0.58 |
|  | **Fremantle score** | | | | | | | | | |
| O'Loughlin et al. 2017 [55] | | post hoc video analysis | - | 25 videos, unknown setting | post hoc Fremantle score rated by 74 critical care physicians | post hoc Fremantle score rated by 4 senior anaesthetists* | no outcome measure | good-Fleiss κ 0.62, Intra-rater 0.80, concordance 74% | not defined | Perf.: not assessed│Acc.: not assessed│Cal.: not assessed |
|  | **Video Classification of Intubation score (VCI)** | | | | | | | | | |
| Chaggar et al. 2021 [36] | | prospective study | - | 35 adults; airway surgery; OR | VCI rated by a trainee performing the intubation^§^ | VCI rated by a consultant performing a laryngoscopy and watching trainee’s intubation^§^ | no outcome measure | Concordance 97% | not defined | Perf.: not assessed│Acc.: not assessed│Cal.: not assessed |
| Halliday et al. 2025 [37] | | prospective multicentre study | - | 1644 paired data sets from adults and children | VCI rated by a staff member performing the intubation^§^ | VCI rated by another staff member watching the intubation^§^ | no outcome measure | good-Krippendorff alpha 0.86, concordance 89% | not defined | Perf.: not assessed│Acc.: not assessed│Cal.: not assessed |
|  | **Videolaryngoscopic intubation and difficult airway classification (VIDIAC) score** | | | | | | | | | |
| Kohse et al. 2022 [11] | | prospective study | development and validation | 320 adults (374 data sets); exp. difficult airways; OR | VIDIAC items rated by 81 anaesthetists +2 independent raters | difficult videolaryngo-scopic intubation | 183/374 (49%) | good-Fleiss’ κ 0.72 to 1.0 for each component | decision curve analysis; 2 pts. “hard”, 3 pts. “severe” VL | Perf.: good-AUROC 0.92 (0.89–0.95)│Acc. (2 pts.): sens. 0.74 (0.65-0.81), spec. 0.95 (0.89-0.98)│Cal.: good (calibration belt) |
| Dohrmann et al. 2024 [39] | | prospective study | external validation | 809 children (904 data sets); OR | VIDIAC rated by anaesthetists^§^ +1 independent rater | difficult videolaryngo-scopic intubation | 47/904 (5%) | not provided | based on prior study [11], recalibration | Perf.: good-AUROC 0.80 (0.73–0.87)│Acc. (0 pts.): sens. 0.68, spec 0.96, │Cal.: good (calibration belt) |
| Köhl et al. 2025 [38] | | prospective study | external validation | 182 adults; exp. difficult airways; OR | VIDIAC rated by 19 consultant anaesthetists | difficult videolaryngo-scopic intubation | 66/182 (36%) | not provided | based on prior study [11], reconfirmation | Perf.: good-AUROC 0.93 (0.88–0.97)│Acc. (2 pts.): sens. 0.82, spec. 0.68, Cal.: good (calibration belt)│validation for different blade types |
|  | **Pediatric Difficult Airway Classification (PeDiAC) score** | | | | | | | | | |
| Dohrmann et al. 2024 [39] | | prospective study | development and validation | 809 children (904 data sets); OR | PeDiAC items rated by anaesthetists^§^ +1 independent rater | difficult videolaryngo-scopic intubation | 47/904 (5%) | not provided | Youden/ utility-based; 1pt. “notable” 2 pts. “difficult” VL | Perf.: good-AUROC 0.97 (0.96–0.99)│Acc. (2 pts.): sens. 0.91 (0.83-0.98), spec. 0.93 (0.91-0.95)│Cal.: good (calibration belt) |

studies that explicitly report at least one test characteristics, such as concordance, reliability, accuracy or performance of a classification were considered; concordance between raters is the proportion (%) of ratings where both raters (or group of raters) agree; a data set refer to the assessed set of data per anaesthesia encounter, TI: tracheal intubation; ICC: intraclass correlation; ^#^originating from a multivariable regression model; ^+^assessed in Bland–Altman plot; ED, emergency department; OR, operation room; ICU, intensive care unit; NICU, Neonatal Intensive Care Unit; *authors of the respective study considered a panel (group of raters) rating being a reference standard for the calculation of reliability, concordance or accuracy, ^§^number of participating airway operators not reported; MAC-VL: Macintosh and HA-VL: hyperangulated videolaryngoscopy (VL); performance (Perf.) is expressed as area under the receiver operating characteristic curve (AUROC); accuracy (Acc.) is given by the sensitivity (sens.), specificity (spec.) of the index test to correctly classify the respective outcome condition

**Table S4** Quality appraisal of included studies that assessed inter-rater reliability using the QAREL checklist.

| **Classification tool** | **Cormack-Lehane** | | | | | | | **POGO** | | **Fremantle** | **VCI** | | **VIDIAC** |
| --- | --- | --- | --- | --- | --- | --- | --- | --- | --- | --- | --- | --- | --- |
| **Items** | Naito [53] | Naito [54] | O'Loughlin [55] | O'Shea [56] | Garcia-Pintos [52] | Unal [51] | Sasu [5] | O'Loughlin [55] | Unal [51] | O'Loughlin [55] | Chaggar [36] | Halliday [37] | Kohse [11] |
| 1. Was the test evaluated in a sample of subjects who were representative of those to whom the authors intended the results to be applied? | U | U | N | U | U | U | Y | N | U | N | Y | N | Y |
| 2. Was the test performed by raters who were representative of those to whom the authors intended the results to be applied? | Y | U | Y | Y | Y | Y | Y | Y | Y | Y | Y | Y | Y |
| 3. Were raters blinded to the findings of other raters during the study? | U | U | Y | Y | U | Y | U | Y | Y | Y | U | U | U |
| 4. Were raters blinded to their own prior findings of the test under evaluation? | N/A | N/A | U | N/A | N/A | U | U | U | U | U | N/A | U | U |
| 5. Were raters blinded to the results of the accepted reference standard or disease status for the target disorder or variable being evaluated? | N/A | N/A | N/A | N/A | N/A | N/A | U | N/A | Y | N/A | N/A | N/A | U |
| 6. Were raters blinded to clinical information that was not indented to be provided as part of the testing procedure or study design? | U | U | U | N | U | Y | U | U | Y | U | U | U | U |
| 7. Were raters blinded to additional cues that were not part of the test? | U | U | N | U | Y | Y | U | N | Y | U | U | U | U |
| 8. Was the order of examination varied? | N/A | N/A | N/A | N/A | N/A | N/A | N/A | N/A | N/A | N/A | N/A | N/A | N/A |
| 9. Was the stability of the variable being measured taken into account when determining the sustainability of the time-interval between repeated measures? | Y | N | Y | Y | Y | Y | U | Y | Y | Y | N | U | U |
| 10. Was the test applied correctly and interpreted appropriately? | Y | U | U | N | U | U | Y | U | Y | U | U | U | Y |
| 11. Were appropriate measures of agreement used? | Y | Y | Y | N | Y | Y | Y | N | Y | Y | N | Y | Y |

Y, yes; N, no; U, unclear; N/A, not applicable

**Table S5** Summary of risk of bias and applicability concerns of multivariable prediction model development and validation studies assessed with the PROBAST instrument.

| **Quality rating** | **Risk of bias** | | | | **Applicability** | | |
| --- | --- | --- | --- | --- | --- | --- | --- |
| **Author, Year** | **Participants** | **Predictors** | **Outcome** | **Analysis** | **Participants** | **Predictors** | **Outcome** |
| **Cormack-Lehane classification** | | | | | | | |
| Kohse et al. 2022 [11] | + | ? | - | ? | + | + | + |
| Sasu et al. 2024 [5] | + | ? | - | ? | + | + | + |
| **Videolaryngoscopic intubation and difficult airway classification (VIDIAC) score** | | | | | | | |
| Kohse et al. 2022 [11] | + | + | - | + | + | + | + |
| Dohrmann et al. 2024 [39] | + | ? | - | + | + | + | + |
| Köhl et al. 2025 [38] | + | ? | - | ? | + | + | + |
| **Pediatric Difficult Airway Classification (PeDiAC) score** | | | | | | | |
| Dohrmann et al. 2024 [39] | + | + | - | + | + | + | + |
